# TREM-1 and TREM-2 Expression on Blood Monocytes Could Help Predict Survival in High-Grade Glioma Patients

**DOI:** 10.1155/2020/1798147

**Published:** 2020-07-01

**Authors:** K. Kluckova, J. Kozak, K. Szaboova, B. Rychly, M. Svajdler, M. Suchankova, E. Tibenska, B. Filova, J. Steno, V. Matejcik, M. Homolova, M. Bucova

**Affiliations:** ^1^Institute of Immunology, Faculty of Medicine, Comenius University, Bratislava, Slovakia; ^2^Department of Neurosurgery, Faculty of Medicine, Comenius University and University Hospital, Bratislava, Slovakia; ^3^Medirex, Ltd., Bratislava, Slovakia; ^4^Alpha Medical, Ltd., Bratislava, Slovakia; ^5^Cytopathos, Ltd., Bratislava, Slovakia; ^6^Sikl's Department of Pathology, Charles University, The Faculty of Medicine and Faculty Hospital in Pilsen, Czech Republic; ^7^Institute of Medical Physics, Biophysics, Informatics and Telemedicine, Faculty of Medicine, Comenius University, Bratislava, Slovakia

## Abstract

**Objective:**

In recent years, the role of the modern inflammatory markers TREM-1 (triggering receptors expressed on myeloid cells) and HMGB1 (high mobility group box 1 protein) in tumorigenesis has begun to be studied. Their role in gliomas is not clear. The aim of our study was to find the role of inflammation in gliomas. *Patients and Methods*. In 63 adult patients with gliomas and 31 healthy controls, the expressions of TREM-1 and TREM-2 on CD14^+^ blood cells (method: flow cytometry) and the levels of soluble sTREM-1, HMGB1, IL-6, and IL-10 (Elisa tests) were analyzed.

**Results:**

Cox proportional hazard analysis showed that a TREM-1/TREM-2 ratio was associated with reduced overall survival (HR = 1.001, *P* = 0.023). Patients with a TREM-1/TREM-2 ratio above 125 survived significantly shorter than patients with a TREM-1/TREM-2 ratio below 125. The percentage of CD14^+^ TREM-1^+^ cells was strongly associated with a plasma IL-6/IL-10 ratio (positively) and with IL-10 (negatively). Conversely, we found a higher percentage of CD14^+^ TREM-2^+^ monocytes in better surviving patients; these cells could downregulate the exaggerated inflammation and potentiate the phagocytosis in the tumor. The serum levels of HMGB1 negatively correlated with the percentage of CD14^+^ TREM-1^+^ cells and with the TREM-1/TREM-2 ratio. The positive correlation between the serum levels of a late proinflammatory cytokine HMGB1 with the percentage of TREM2^+^ CD14^+^ monocytes can be explained as an effort for suppression of systemic inflammation by anti-inflammatory acting CD14^+^ TREM-2^+^ cells.

**Conclusion:**

We showed that the TREM-1/TREM-2 ratio (expression on the surface of blood monocytes) could help predict prognosis in patients with gliomas, especially in high-grade gliomas, and that systemic inflammation has an impact on the patient's overall survival. This is the first study that showed that TREM expression on monocytes in peripheral blood could help predict prognosis in patients with gliomas.

## 1. Introduction

Gliomas belong to the most frequent primary brain tumors with diverse pathological and histological properties. More than half of patients present a diagnosis of glioblastoma (GBM, grade IV), the most aggressive and lethal form of glioma. Despite an improvement in understanding glioma biology, this disease still has bad prognosis. In 2012, the median survival period of the most common form of GBM was approximately 15 months [1]. In 2019, Cantrell et al. showed that despite improvements in the median and short-term overall survival, the percentage of patients with glioblastoma achieving 5-year overall survival remains very low at -4.6% [2]. The 10-year survival rate was estimated by Tykocki and Eltayeb to be 0.71% [3].

Antitumor immunity was initially considered to be a part of immune surveillance—the ability of the immune system of the host to recognize abnormal cells and to destroy them. Today, the hypothesis of “cancer immunoediting,” immunocorrection, is preferred. The basis of this hypothesis is that the immune system in addition to protecting the host from tumor formation also “shapes” the immunogenicity of tumor cells, which leads to the change of strong immunogenic tumor-specific antigens to weak ones [4]. Inflammation plays a great role in this process and is considered one of the hallmarks of cancer [5]. However, its role in gliomas is less clear than that in other types of cancer.

Inflammation develops as a defense mechanism to any damage caused by infectious agents, tissue injury, or malfunction [6]. The acute inflammation is beneficial, helping to eliminate infectious agents, tumor cells, and foreign substances and promoting tissue repair. The acute inflammation accompanies also the first phase of immunoediting (phase of tumor elimination) where it blocks the tumor formation. Once the immune response has neutralized the threat, the proinflammatory molecules are replaced by anti-inflammatory ones. However, if the cause of the initial inflammatory response is not resolved, the acute inflammation can shift to a chronic one [7–10].

Both systemic and local inflammation in the tumor microenvironment can support cancer development. Under the physiological state, the only cells with immune functions in the central nervous system (CNS) are microglia. However, after blood brain barrier (BBB) disruption induced by trauma, stress, or other pathological conditions, the proinflammatory molecules and immune cells from the periphery can cross the BBB and enter the CNS [11]. As gliomas can also lead to BBB disruption, circulating immune cells not normally found in the CNS—macrophages, various types of T and B cells, regulatory T cells (Tregs), and myeloid-derived suppressor cells (MDSCs)—gain access to tumor areas [12, 13].

Glioma-associated microglia that account for 30% of the glioma mass tend to secrete cytokines and growth factors that promote tumor cell growth and angiogenesis. Many of these cytokines and molecules related to inflammation and secreted from tumor and immune cells are connected with tumorigenesis [14].

In recent years, the role of the modern inflammatory markers TREM-1 (triggering receptors expressed on myeloid cells) and HMGB1 (high mobility group box 1 protein) in tumorigenesis has begun to be studied. Their role in gliomagenesis is not explained. The expression of TREM-1 receptor is associated with activated Th1 cell-mediated immunity, which is associated with antitumor immunity during the initiation phase of tumor growth. However, the long-lasting presence of this molecule supports the proinflammatory state at both systemic and local levels directly in the tumor microenvironment (TME), where it potentiates the tumor growth [15–17]. Future studies will establish the role of TREM-1 in tumor growth and define the mechanisms by which TREM-1 modulates tumor growth.

Soluble TREM-1 as a form of TREM-1 serves as a decoy receptor; binding its ligands has anti-inflammatory activity [6, 18]. Elevated serum/plasma levels of sTREM-1 were found in inflammatory diseases of both infectious and noninfectious origins [18, 19]. Both increased and decreased serum levels were detected in some types of cancers [20].

HMGB1 is an intranuclear protein with a lot of vital intranuclear functions. Passively released by necrotic cells or actively produced by activated monocytes, macrophages, and tumor cells, HMGB1 enters to the extracellular space and exerts there its proinflammatory activities. Extracellular HMGB1 serves as an alarmin or a damage-associated molecular pattern (DAMP) and acts as a late proinflammatory cytokine [21]. It also promotes cell survival and cell death by regulating multiple signalization pathways [22]. Extracellular HMGB1 activates proinflammatory signals and supports tumorigenesis, thereby promoting inflammation, progression of the tumor, and formation of metastases [22]. Contrary, in breast cancer, intracellular HMGB1 acts as an inhibitor of tumor progression while binding Rb (retinoblastoma) protein [23]. There is an interconnection between TREM-1 and HMGB1 molecules, because HMGB1, besides binding to its own specific receptor, serves also as a ligand for the TREM-1 receptor [24, 25]. However, it appears that HMGB1 needs coactivating molecules for TREM-1 stimulation [26]. Immune cells that infiltrate the tumor and cytokine signal pathways are essential to preserving the inflammatory tumor microenvironment (TME) [6].

TREM-2 is a negative regulator of inflammatory response. It is expressed in different tissues on dendritic cells, peritoneal and pleural macrophages, and microglia [27]. Its expression on microglia is well known, but the exact role and signal pathways are the objects of further investigation [27, 28]. The TREM-2 molecule has an anti-inflammatory effect, promotes phagocytosis, and is associated with Th2 immunity and cell-mediated immune suppression that could potentiate the tumor growth [29].

Based on the knowledge that chronic systemic low-grade inflammation plays a great role in tumorigenesis, the aim of our study was to find the possible negative role of new inflammatory markers TREM-1 and HMGB1 on the prognosis of malignant gliomas of Slovak patients. We detected the expression of TREM-1 and TREM-2 receptors on the surface of CD14-positive blood cells and the level of soluble factors sTREM-1 and HMGB1 in the serum, and for better understanding, we also determined the level of proinflammatory cytokine IL-6 and anti-inflammatory cytokine IL-10. We wanted to know if these molecules could serve as markers of diagnosis or prognosis in cancer patients. We tried to find a correlation between these markers and the survival rate, the tumor grade, and other laboratory parameters and to explain their role in the diagnosis and prognosis of glioma patients.

## 2. Subjects and Methods

The study group included 63 patients older than 18 years with partial or complete resection of CNS tumor. Patients with primary diagnosis and with relapse of tumor were analyzed. In our cohort, only patients with histologically proven glioma grades II, III, and IV were enrolled; all other histological types of tumors or other diagnoses were excluded. Tumors of grade II with signs of grade III were taken as grade III, and one tumor of grade III with signs of glioblastoma was taken as grade IV. The diagnosis was approved by two neuropathologists according to the most recent WHO classification criteria. Blood samples were obtained from the patients at one day before surgical treatment.

The reference cohort in our case-control study comprised of 31 unrelated volunteers for flow cytometry analysis (14 males and 17 females from 26 to 75 years) and 23 of them for ELISA tests. All control subjects were without any personal or family history of gliomas, and they were randomly recruited from a larger population sample. All patients and controls were Caucasians of Slovak descent. All the investigations were carried out in accordance with the International Ethical Guidelines and the Declaration of Helsinki. Written informed consent for enrolling in the study and for personal data management was obtained from all examined cases. The study was approved by the Ethical Committee of University Hospital in Bratislava.

The blood was obtained between the years 2015 and 2018. The percentage of CD14^+^ monocytes (Mo) and TREM-1 and TREM-2 expressions on CD14^+^ monocytes were measured by flow cytometry (Navios, Beckman Coulter France S.A.S.). Both the percentage and MFI (mean fluorescence intensity) of TREM expressions were analyzed by KALUZA analysis software (Beckman Coulter France S.A.S.) (antibodies used: CD14-PC7, TREM-1-PE, TREM-2-APC, and isotype controls; all from R&D System, Minneapolis, MN, USA). The TREM-1 and TREM-2 analysis that we used in our previous studies [30, 31] was performed in compliance with flow cytometry protocol recommended by the manufacturer. TREM-1 and TREM-2 expression is presented as the percentage of TREM-1- and TREM-2-positive cells out of all CD14^+^ cells. For each patient, we performed a negative control, sample stained with CD14, and isotype controls without TREM-1 and TREM-2 antibody. The serum levels of soluble sTREM-1 and HMGB1 and plasma levels of IL-6 and IL-10 were analyzed by a sandwich ELISA test (Human sTREM-1 Elisa test; Human HMGB1 Elisa test; Human IL-6 Elisa test; Human IL-10 Elisa test; all from Fine Tests, China) precisely according to the instruction of the manuscripts. The absolute count of monocytes was determined by an automated hematology analyzer (Sysmex XN 1000, Sysmex, Japan). For better specification of inflammation, we also determined the TREM-1/TREM-2 and IL-6/IL-10 ratios. Survival time was calculated from the time of diagnosis until April 2019 or the time of death. Patients were monitored from 1 of December 2015 to 30 of April 2019.

### 2.1. Statistics

For statistical analysis, we used programs InStat and SAS. We used Student's *t*-test, Mann–Whitney test, Cox proportional hazard analysis, Kaplan-Meier survival analysis, Log Rank test, and Spearman correlation. The results were expressed as the median and interquartile range (IQR), mean ± standard deviation (SD), and hazard ratio (HR). *P* value < 0.05 was considered to indicate statistical significance.

## 3. Results

### 3.1. Basic Characteristics of Glioma Patients Are Summarized in Table 1

### 3.2. Percentage of CD14^+^ Monocytes in Glioma Patients and Healthy Controls

The difference in the percentage of CD14^+^ Mo between all groups of glioma patients and healthy controls was not statistically significant. However, patients with GBM-G. IV had a statistically significantly lower percentage of CD14^+^ monocytes than G. II gliomas (Student's *t*-test; mean 7.0 ± 2.32*vs*. 4.69 ± 2.11; *P* = 0.001). This difference was not observed in absolute monocyte count. The difference between GBM and healthy controls was also statistically significant (Student's *t*-test; mean 4.69 ± 2.11*vs*. 6.03 ± 2.51; *P* = 0.031).

### 3.3. Percentage of CD14^+^ TREM-1^+^ and CD14^+^ TREM-2^+^ Monocytes and TREM-1/TREM-2 Ratio in Glioma Patients and Correlations with Survival

The range of CD14^+^ TREM-1^+^ Mo in healthy subjects was from 15.85% to 80.8% (mean 44.33%; median 41%), the percentage of CD14^+^ TREM-2^+^ Mo ranged from 0% to 16% (mean 1.39%; median 0.25%), and TREM-1/TREM-2 ratio was from 1.23 to 1042 (mean 261.77; median 169.57). We did not find significant differences when comparing TREM-1 and TREM-2 expressions on monocytes between glioma grades and between gliomas and healthy controls.

**Figure 1:**
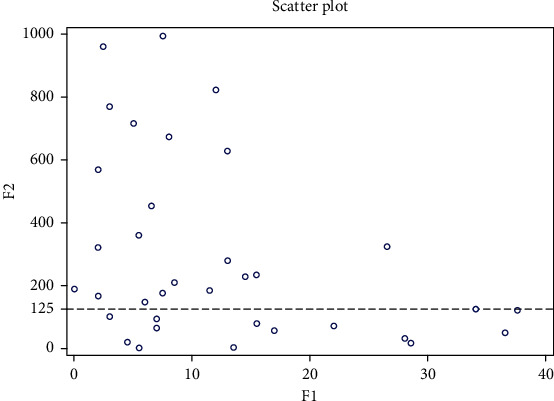
Scatter plot of correlation between survival time and TREM-1/TREM-2 ratio in grades III and IV (*N* = 37). F1: survival time in months; F2: TREM-1/TREM-2 ratio.

When we performed Cox hazard proportional analyses separately for each parameter, the TREM-1/TREM-2 ratio was proven to have influence on overall survival time (HR = 1.001, *P* = 0.023) (Table 2). We found a significant correlation between the TREM-1/TREM-2 ratio and overall survival time in high-grade glioma patients. Survival time is reduced with a TREM-1/TREM-2 ratio higher than 125, as shown in Figure 1. However, we did not observe the correlation of the overall survival time with TREM-1 or TREM-2 separately.

**Figure 2:**
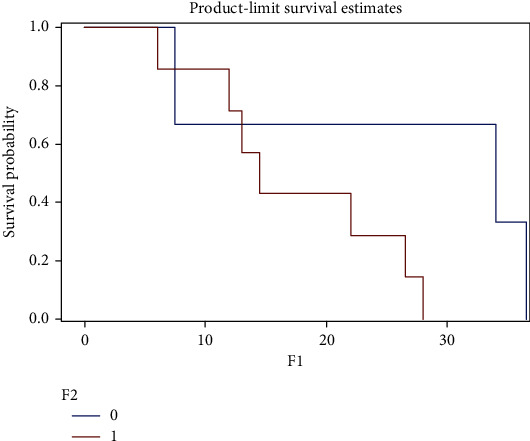
Kaplan-Meier survival curves for grade III gliomas. The graph shows a shorter survival in patients with the TREM-1/TREM-2 ratio > 125 (*P* = 0.012) (test used: Log Rank; *x*-axis: survival time in months; *y*-axis: survival probability; 0: patients with TREM-1/TREM-2 ratio < 125 (*n* = 4); 1: patients with TREM-1/TREM-2 ratio > 125 (*n* = 6)).

**Figure 3:**
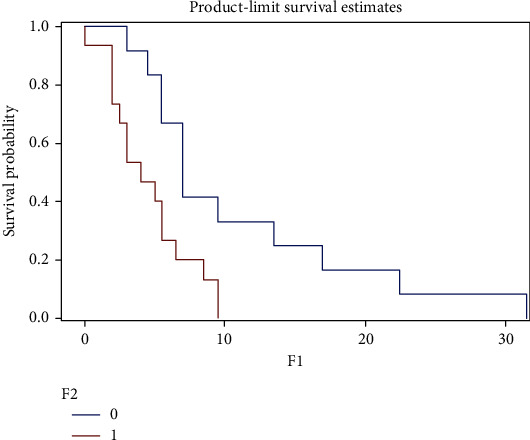
Kaplan-Meier survival curves of grade IV gliomas—GBM. The graphs shows a shorter survival in patients with TREM-1/TREM-2 ratio > 125 (*P* = 0.007). Test used: Log Rank; *x*-axis: survival time in months; *y*-axis: survival probability; 0: patients with TREM-1/TREM-2 ratio < 125 (*n* = 12); 1: patients with TREM-1/TREM-2 ratio > 125 (*n* = 15); GBM: glioblastoma multiforme—grade IV.

We tried to determine two groups of patients with significantly different overall survival times according to the TREM-1/TREM-2 ratio. We found the cut-off value for the TREM-1/TREM-2 ratio as 125. We performed Kaplan-Meier survival curves for grades III and IV (Figures 2 and 3) (Log Rank test, *P* = 0.012 and *P* = 0.007, resp.)

The mean survival time in G. IV was 11.6 months in the better surviving group *vs*. 5.8 in the worse surviving group. In G. III, the mean survival time was 31.1 for the better surviving group *vs*. 13.3 months in the worse surviving patients. In mean fluorescence intensity (MFI), we did not find any differences between glioma grades or between patients and healthy controls nor correlations with survival. We did not find any correlation between event-free survival (as an event, we determined time of relapse, death, or end of observation) and TREM-1, TREM-2, or TREM-1/TREM-2 ratio. We had 30 patients with grades IV and 13 with grade III, but in some patients, the flow cytometry measurements were not available. This is the reason why the numbers of patients are different between survival analyses.

### 3.4. Percentage of CD14^+^ TREM-2^+^ Monocytes in Better and Worse Surviving Patients in the GBM Subgroup

**Figure 4:**
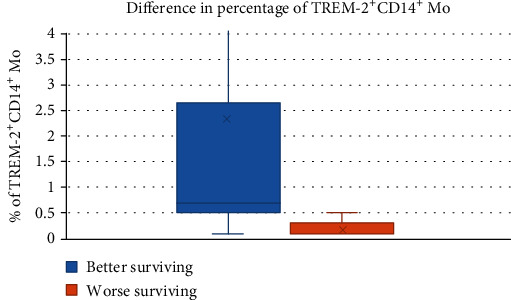
Percentage of CD14+ TREM-2+ monocytes in good and bad surviving patients in GBM – G. IV patients. Pts: patients.

We found a higher percentage of CD14^+^ TREM-2^+^ cells in better surviving patients than in worse surviving patients in the GBM subgroup (Mann–Whitney test; median 0.55, IQR 1.675 *vs*. median 0.10, IQR 0.3; *P* = 0.013) (Figure 4).

### 3.5. The Serum Levels of sTREM-1 and HMGB1 in the Group of All Glioma and the Subgroup of GBM Patients

**Figure 5:**
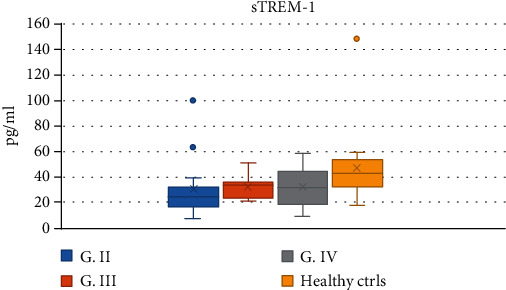
The serum levels of sTREM-1 in glioma patients and healthy controls. G: grade; ctrls: controls.

**Figure 6:**
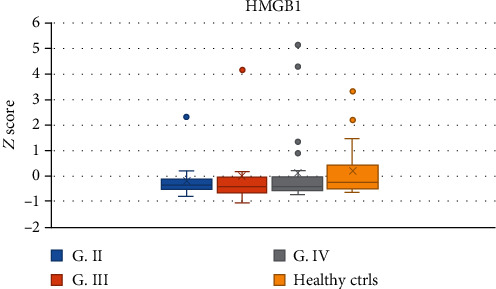
The serum levels of HMGB1 in glioma patients and healthy controls. G: grade; ctrls: controls.

The serum levels of sTREM-1 in the group of all glioma patients as well as in the GBM subgroup were significantly lower than those in healthy controls (Mann–Whitney tests; all gliomas median: 29.96 pg/mL, IQR 18.793; *vs.* healthy controls median: 42.95 pg/mL, IQR 20.875; *P* = 0.001; G IV median: 31.58 pg/mL, IQR 25.415 *vs.* healthy controls median 42.95 pg/mL, IQR 20.875; *P* = 0.013, resp.) (Figure 5). There was no statistically significant difference in serum levels of HMGB1 between glioma grades and between glioma patients and healthy controls (values corrected by a *Z*-score) (Figure 6).

### 3.6. Correlations between HMGB1 and TREM Molecules in All Glioma Patients and the GBM Subgroup

**Figure 7:**
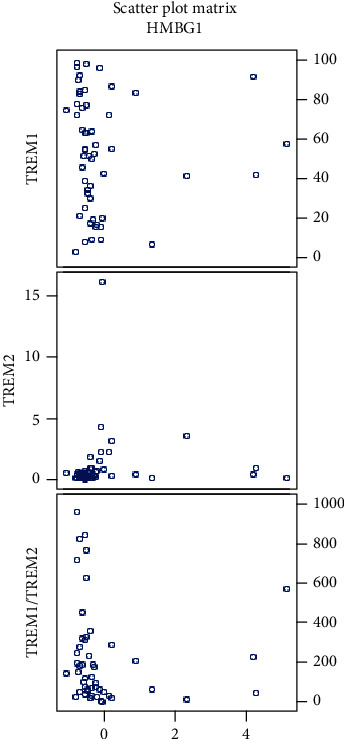
Scatter plots showing correlations of HMGB1 with percentage of CD14^+^ TREM-1^+^ cells, CD14^+^ TREM-2^+^ cells, and TREM-1/TREM-2 ratio in all gliomas (HMGB1 values corrected by a *Z*-score).

We found a negative correlation of the serum levels of HMGB1 in glioma patients with both percentages of CD14^+^ TREM-1^+^ cells (*P* = 0.049) and TREM-1/TREM-2 ratio (*P* = 0.004) (Table 2). Very interesting was the finding of a positive correlation of the level of HMGB1 in glioma patients with percentage of CD14^+^ TREM-2^+^ Mo (*P* = 0.007) (Table 3, Figure 7).

**Figure 8:**
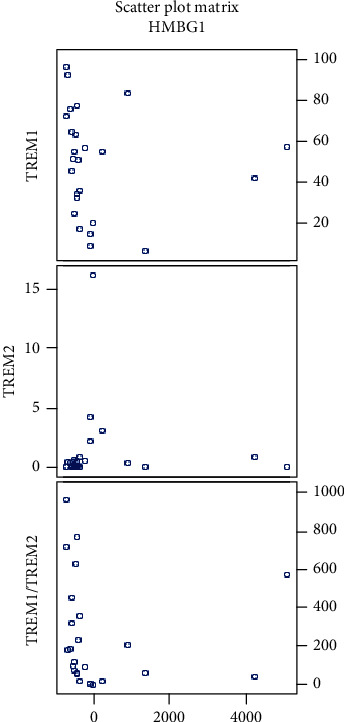
Scatter plots showing correlations of HMGB1 with percentage of CD14^+^ TREM-1^+^ cells, CD14^+^ TREM-2^+^ cells, and TREM-1/TREM-2 ratio in grade IV gliomas (HMGB1 values corrected by a *Z*-score).

These correlations were also observed in the subgroup of GBM patients; the negative correlation of HMGB1 with the percentage of CD14^+^ TREM-1^+^ cells had a *P* value of 0.016 and for negative correlation of HMGB1 with the TREM-1/TREM-2 ratio, the *P* value was 0.008 (Table 2, Figure 8). We did not find these correlations in grades II and III separately.

Regarding corticosteroids therapy, we did not find any significant difference in TREM-1 and TREM-2 expressions or serum levels of sTREM-1 and HMGB1, but we found a significantly lower percentage of CD14^+^ Mo in steroid-treated patients than in nonsteroid-treated patients. In G. II, the *P* value was 0.041 (mean 7.873 ± 2.006*vs*. 5.629 ± 2.225), in G. III, the *P* value was 0.069 (mean 7.400 ± 1.825*vs*. 4.971 ± 1.619), and in G. IV, the *P* value was 0.006 (mean 7.7 ± 2.117*vs.*4.332 ± 1.835).

### 3.7. Correlation of TREM-1 Expression with Plasma Levels of IL-6 and IL-10

**Figure 9:**
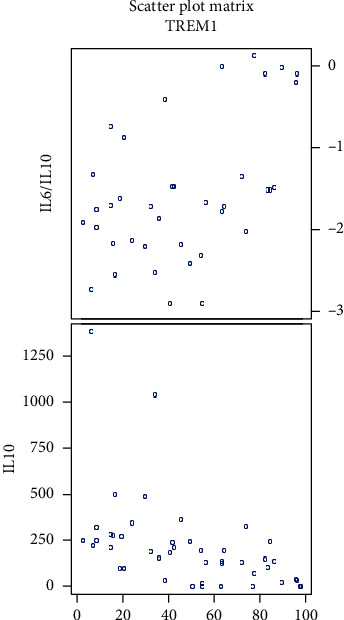
Scatter plots showing correlations of CD14^+^ TREM-1^+^ cell percentage with IL-6/IL-10 ratio and with IL-10 in all gliomas (data log transformed).

**Figure 10:**
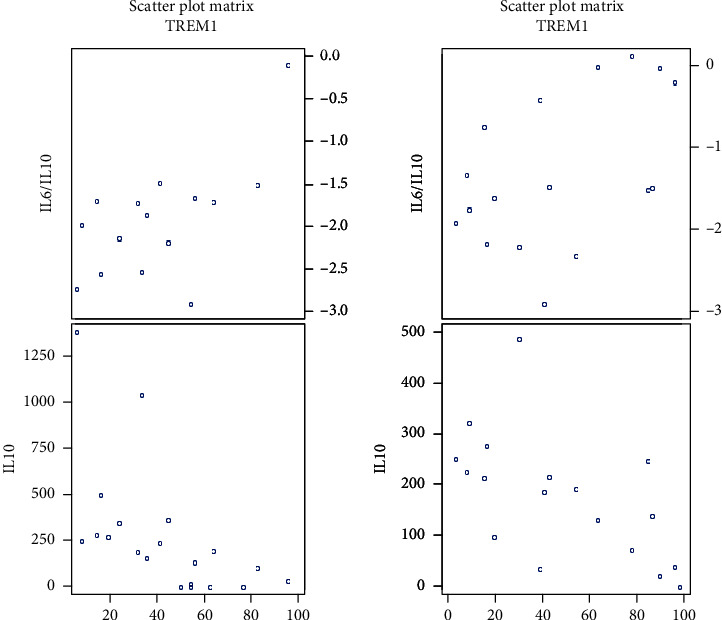
Scatter plots showing correlations of CD14^+^ TREM-1^+^ cell percentage with IL-6/IL-10 ratio and with IL-10 in grade II (on the left side) and in grade IV (on the right side) (data log transformed).

In all glioma patients, we observed a positive correlation of percentage of CD14^+^ TREM-1^+^ cells with the IL-6/IL-10 ratio (*P* = 0.007) and a negative correlation with the plasma levels of IL-10 (*P* < 0.0001). Strong correlations were seen in glioma grades II and IV. Correlations in all gliomas and different grades are summarized in Table 4; scatter plots are shown in Figures 9 and 10.

### 3.8. TREM-1 and TREM-2 Expression on Monocytes according to Age

When we compared patients of different age groups, we did not observe any differences in TREM-1 or TREM-2 expression.

## 4. Discussion

In 1858, Virchow demonstrated the interconnection of inflammation with cancer development [25]. An inflammatory process has a role in all phases of tumorigenesis—in initiation, promotion, and progression, and immune cells and mediators can contribute to tumor growth during chronic inflammation [32–34].

We found a significantly lower percentage of monocytes in the peripheral blood in G. IV glioma patients in comparison with G. II glioma patients and healthy controls. This could be due to the larger proportion of monocytes migrating into the tumor in high-grade gliomas. The phenomenon of the high infiltration of tumor-associated macrophages (TAMs) into GBMs has been observed for a long time. One of the most commonly detected phenomena in GBM is the abundant macrophage infiltration without apparent phagocytic activity [35]. There was a significant increase in the number of macrophages in high-grade astrocytomas, *i.e.*, GBMs [36]. Other studies showed a strong correlation of macrophages with tumor malignancy [37]. Compelling evidence in several different solid tumors indicated that tumor-associated macrophages come from monocyte recruitment [38]. However, the situation in brain tumors is more complex. This is largely due to the unique immune cells, *i.e.*, microglia, which exclusively reside in the brain. Mature microglia share a variety of cell surface markers with monocyte-derived macrophages; therefore, it is hard to distinguish microglia from macrophages in GBM tumors [6].

A higher TREM-1/TREM-2 ratio was associated with shorter overall survival in grades III and IV. In G. IV, only 2 patients from the better surviving group and 2 patients from the worse surviving group had completely resected tumors. In G. III, there was 1 patient with completely resected tumor in each group. We believe that this factor, which is considered prognostically favorable, did not influence the significance of our results. There are two possible explanations for our findings. (1) We suppose that an increased TREM-1/TREM-2 ratio in blood potentiates the systemic inflammation that plays a great role in all phases of tumor growth and progression. Taken together, both local and chronic systemic low-grade inflammation might account for the shorter survival rate of patients. (2) Moreover, TREM-1^+^ monocytes from the peripheral blood enter into gliomas, where they promote inflammation. The chronic inflammation supports tumor growth, and this could be the reason why these patients survived a shorter time.

TREM-1 expression was described on TAMs (tumor-associated macrophages (TAMs)) and was also associated with shorter survival in other cancer types. This was demonstrated in nonsmall lung cell carcinoma (NSLCC) [20]. However, Zhang et al. confirmed that with the progression of NSLCC, the expression of TREM-1 on TAMs was decreasing. They compared the expression of TREM-1 on monocytes in the peripheral blood, and they found that the expression of TREM-1 on macrophages in the tumor was lower than on monocytes in the peripheral blood so the condition in the peripheral blood appears to be opposed to the tumor. This could suggest enhanced effort in the peripheral blood to fight against a growing tumor [20]. Wu et al. in 2012 demonstrated that TREM-1 is a key determinant of Kupffer cell activation in hepatocellular carcinoma (HCC) [39]. The inhibition of the TREM-1 signaling pathway could suppress the growth of HCC, and it could be a potential treatment target [39]. Duan et al. demonstrated TREM-1 expression on tumor cells in HCC. Furthermore, they found a correlation of TREM-1 expression with tumor recurrence and bad prognosis [40]. Anaya-Prado et al. in 2015 found high TREM-1 expression on macrophages in advanced cervical carcinomas and compared it with premalignant tissue samples [41]. Saurer et al. pointed out the clear role of TREM-1 in colorectal cancer development [42]. Nguyen et al. in 2015 made a finding that the TREM-1 and TREM-2 ratio better indicated the intensity of inflammation in tumor tissue than TREM-1 or TREM-2 expression alone [43]. This statement also supports our results. Yuan et al. in 2014 published a study in which they demonstrated that the expression of TREM-1 is increased in macrophages that are cocultured with human lung cancer cells [44].

Our results showed that patients with GBM with better overall survival had a significantly higher percentage of CD14^+^ TREM-2^+^ monocytes in the peripheral blood. We suppose that TREM-2^+^ monocytes arise as a contraregulatory subpopulation of cells with the effort to downregulate the exaggerated inflammation—both systemic and local—and potentiate the phagocytosis in the brain tumor.

Some papers demonstrated the function of TREM-2 in tumorigenesis. While some studies found the protective role of TREM-2 in cancer, others showed its protumorigenic potential. In 2016, Wang et al. clearly demonstrated the overexpression of TREM-2 protein in glioma tumor samples in comparison with nontumor tissues. The expression was higher in high-grade gliomas in comparison to low-grade gliomas, and the expression negatively correlated with the patient's survival. They supposed it functions as an oncogene [45]. The same year, Yao et al. described higher expression of TREM-2 on monocytes in patients with lung carcinoma than in healthy controls. Patients had more TREM-2 positive macrophages in lung tumors than patients with benign lung diseases. The amount of TREM-2 receptors on macrophages positively correlated with TNM (tumor, nodes, and metastasis) stage [46]. Zhang et al. in 2018 described a negative correlation of TREM-2 expression and overall survival in gastric cancer tissues. TREM-2 protein and its mRNA expression were higher in higher TNM stages with a worse prognosis [47]. Zhang et al. showed high TREM-2 expression in renal cell carcinoma. Knockdown of TREM-2 inhibited cell growth and induced cell cycle arrest and apoptosis of tumor cells [48]. On the contrary, in a recent study from 2019, Tang et al. described TREM-2 as a tumor suppressor. They found low TREM-2 protein expression in human HCC samples. This low expression correlated with shorter survival time. Knockdown of TREM-2 induced cell proliferation, migration, and increased invasive potential “*in vivo*” and “*in vitro*,” while TREM-2 overexpression had the opposite effect. TREM-2 also retarded the growth of metastases of HCC [49].

In our cohort of glioma patients, we found that the serum levels of HMGB1 negatively correlated with the percentage of TREM-1^+^ Mo and with the TREM-1/TREM-2 ratio. The negative correlation of the serum level of HMGB1 with the percentage of TREM-1^+^ Mo could be explained by the binding of HMGB1 to the TREM-1 receptor. Moreover, we expect that HMGB1 released from brain tumor necrotic cells or produced by tumor-associated macrophages can recruit TREM-1^+^ monocytes into the tumor.

The positive correlation between the serum levels of a late proinflammatory cytokine HMGB1 with the percentage of TREM-2^+^ CD14^+^ monocytes can be explained as an effort for suppression of systemic inflammation by anti-inflammatory acting CD14^+^ TREM2^+^ cells. The role of HMGB1 in cancer development is unclear; both protumorigenic and antitumorigenic effects are considered depending on many other factors [22].

Various studies investigate the role of HMGB1 in glioma tissue. In our study, we try to explain its role in the peripheral blood. In 2016, Gu et al. published a study where they found that miR-218 (microRNA-218) may negatively regulate HMGB-mediated suppression of RAGE and thereby regulate cell proliferation, apoptosis, and invasion in glioma cells. Transfection of miR-218 significantly reduced the cell viability and colony formation, increased cell apoptosis, and arrested cell in G0/G1 phase. Transfection of miR-218 also decreased the invasion and migration of glioma cells. The expressions of HMGB1, RAGE, cyclin D1, and MMP-9 were downregulated, while the expression of caspase-9 was upregulated by miR-218 [50]. Cheng et al. in 2018 found that HMGB1 is an independent prognostic biomarker for unfavorable prognosis of patients with GBM. HMGB1 released from GBM cells can activate AKT and ERK signaling pathways and promote GBM cell invasion in this autocrine pathway [51]. Contrary, Jia et al. in 2019 found that overexpression of HMGB1 is not associated with the malignancy and outcome of glioma [52].

Patients with gliomas had significantly lower serum levels of sTREM-1 than healthy controls. We think that having a smaller amount of this decoy anti-inflammatory receptor supports the systemic inflammation. There are few studies concerning serum sTREM-1 levels in cancer patients. In a study from 2008, Karapanagiotou et al. observed high levels of sTREM-1 in 50% of breast cancer patients, 33.3% of small cell lung carcinoma (SCLC), 26.7% of colorectal cancer, and 13.3% of non-small-cell lung carcinoma patients (NSCLC). Higher concentrations were observed in the absence of lung metastases [53]. In 2018, Kuemmel et al. published a study in which they demonstrated that sTREM-1 was a marker of short survival in patients with NSCLC. Serum levels of sTREM-1 were not associated with metastasis at the time of diagnosis and were not a predictor of subsequent metastasis. In SCLC patients, sTREM-1 levels were lower than that in NSCLC patients and did not predict survival [54]. In 2008, Huang et al. compared values of sTREM-1 in pleural effusions from various diagnoses. They found significantly lower concentrations of sTREM-1 in the malignant, tuberculous, and transudate groups than in bacterial effusions [55].

The TREM-1^+^ Mo and TREM-1/TREM-2 ratio in the whole cohort positively correlated with the IL-6/IL-10 ratio, which might be explained by the findings that the production of both IL-6 and IL-23 is increased by the activation of TREM-1 [56, 57]. Both IL-6 and IL-23 have been shown to be involved in immunoediting in carcinoma microenvironments and associated with poor prognosis [52–55]. Although speculative, the exaggerated production of these and other mediators through TREM-1 activation may represent the mechanism by which expression of TREM-1 on TAMs can promote tumor growth and progression.

Conversely, the percentage of the TREM-1^+^ Mo and TREM-1/TREM-2 ratio negatively correlated with the plasma levels of IL-10. In 2016, Yao et al. demonstrated that TREM-2 might act as a negative immunoregulatory molecule through the Syk (spleen tyrosine kinase) pathway in an IL-10-dependent manner and partially predict prognosis in patients with lung cancer [46]. The role of IL-10 in cancer is controversial. It is considered immunosuppressive, so it could promote tumor growth. On the other hand, it suppresses excessive inflammation, so it may inhibit the development of the tumor. The concentration of IL-10 in the blood as well as in tumor microenvironment would be crucial in the process of tumorigenesis.

We realize that our study has some limitations. Various histological subtypes of gliomas exist, and the biological behavior of each subtype could influence the processes in the peripheral blood. Moreover, comorbidities of patients may also have an impact on the state of systemic inflammation. In our study, we had both relapsed and primary diagnosed patients, and the inflammatory process could be different in these subgroups because relapsed patients were treated previously.

## 5. Conclusion

TREM molecules play a great role in gliomas. While TREM-1 potentiates the Th1 immunity and inflammation, TREM-2 has contraregulatory activity. At the beginning of tumor formation, this TREM-1 immunostimulatory activity has a positive function—it serves as an antitumor immune mechanism. After a longer time, the Th1 immunity is downregulated by the immunosuppressive activity of growing tumor cells so the antitumor activity of TREM-1 is diminished. However, its proinflammatory activity still persists and supports tumor growth. In our study, we showed that patients with a TREM-1/TREM-2 ratio above 125 (G III and G IV) survived significantly shorter time than other patients. TREM-1 expression on monocytes was strongly associated with plasma levels of IL-6 and IL-10; we showed that systemic inflammation has an impact on a patient's overall survival. Moreover, TREM-1^+^ monocytes could enter into the tumor and promote its growth. We found a higher percentage of CD14^+^ TREM-2^+^ monocytes in better surviving patients; these cells could downregulate the exaggerated inflammation and potentiate the phagocytosis in the tumor. The serum levels of HMGB1 negatively correlated with the percentage of CD14^+^ TREM-1^+^ blood cells and with the TREM-1/TREM-2 ratio. This could be explained by the binding of HMGB1 to the TREM-1 receptor. Moreover, HMGB1 released from brain tumor necrotic cells or produced by tumor-associated macrophages can recruit TREM-1^+^ monocytes into the tumor. The positive correlation between the serum levels of a late proinflammatory cytokine HMGB1 with the percentage of TREM2^+^ CD14^+^ monocytes can be explained as an effort for suppression of systemic inflammation by anti-inflammatory acting CD14^+^ TREM-2^+^ cells.

This is the first study that showed that TREM expressions on monocytes in the peripheral blood could help predict the prognosis of glioma patients.

## Figures and Tables

**Table 1:** Characteristics of glioma patients.

Patients	No.	Mean age ± SD
All gliomas	63	53.29 ± 14.98
Sex (male/female)	38/25	
Grades (male/female)		
G. II	14/5	14/5 (40.47 ± 12.30)
G. II-III	2/0	2/0 (30.5)
G. III	7/4	7/4 (48.55 ± 12.57)
G. III-IV	0/1	0/1 (55)
G. IV	15/15	15/15 (64.07 ± 8.70)
Primary diagnosis	49	
Relapse	13	
Unknown	1	
Diagnosis		
Diffuse glioma II	4	
Oligodendroglioma II	7	
Oligoastrocytoma II	1	
Astrocytoma II	7	
Oligodendroglioma II-III	1	
Astrocytoma II-III	1	
Anaplastic astrocytoma	11	
Anaplastic astrocytoma with signs of GBM	1	
Primary GBM	28	
Unknown GBM	2	
Completely resected		
G. II	4	
G. II-III	0	
G. III	3	
G. III-IV	0	
G. IV	4	
IDH1/2 mutated	25	
G. II, III	24	
G. IV	1	
Steroid treated/untreated		
G. II	7/12	
G. III	9/4	
G. IV	28/3	

No.: number of patients; G.: grade; GBM: glioblastoma multiforme; IDH: isocitrate dehydrogenase.

**Table 2:** Cox proportional hazard analysis of TREM expression and survival time in high-grade gliomas.

Parameter		Parameter estimate	Standard error	Chi-square	*P*	Hazard ratio
TREM-1/TREM-2	Survival time in months	0.00141	0.0006218	5.177	0.023	1.001
TREM-1	Survival time in months	0.00591	0.00686	0.742	0.389	1.006
TREM-2	Survival time in months	0.02557	0.07822	0.107	0.744	1.026

**Table 3:** Correlations of the serum levels of HMGB1 with percentage of CD14^+^ TREM-1^+^, CD14^+^ TREM-2^+^ monocytes, and TREM-1/TREM-2 ratio.

HMGB1 (all gliomas)				95% CI
	CD14^+^ TREM-1^+^ Mo	Spearman *r*	-0.272	
		*P*	0.049	-0.512 to 0.006
	CD14^+^ TREM-2^+^ Mo	Spearman *r*	0.369	
		*P*	0.007	0.102 to 0.587
	TREM-1/TREM-2 ratio	Spearman *r*	-0.398	
		*P*	0.004	-0.611 to -0.132
HMGB1 (G. IV)				
	CD14^+^ TREM-1^+^ Mo	Spearman *r*	-0.479	
		*P*	0.016	-0.741 to -0.091
	CD14^+^ TREM-2^+^ Mo	Spearman *r*	0.363	
		*P*	0.075	-0.050 to 0.670
	TREM-1/TREM-2 ratio	Spearman *r*	-0.516	
		*P*	0.008	-0.762 to -0.140

GBM: glioblastoma multiforme.

**Table 4:** Correlations of the percentage of CD14^+^ TREM-1^+^ blood cells with IL-6/IL-10 ratio and plasma level of IL-10.

% of CD14^+^TREM-1^+^ cells				95% CI
G. II	IL-6/IL-10	Spearman *r*	0.422	
		*P*	0.092	-0.090 to 0.757
	IL-10	Spearman *r*	-0.678	
		*P*	0.002	-0.873 to -0.295
G. III	IL-6/IL-10	Spearman *r*		
		*P*	Non *sg.*	
	IL-10	Spearman *r*		
		*P*	Non *sg*.	
G. IV	IL-6/IL-10	Spearman *r*	0.535	
		*P*	0.033	0.038 to 0.820
	IL-10	Spearman *r*	-0.740	
		*P*	0.0002	-0.894 to -0.430
All gliomas	IL-6/IL-10	Spearman *r*	0.427	
		*P*	0.007	0.119 to 0.660
	IL-10	Spearman *r*	-0.623	
		*P*	<0.0001	-0.779 to -0.396

G: grade; IL: interleukin; Non *sg*: nonsignificant.

## Data Availability

The datasets used and/or analysed during the current study are available from the corresponding author on reasonable request.
